# Natural gas of radiolytic origin: An overlooked component of shale gas

**DOI:** 10.1073/pnas.2114720119

**Published:** 2022-04-04

**Authors:** Maria Naumenko-Dèzes, Wolfram Kloppmann, Michaela Blessing, Raphaël Bondu, Eric C. Gaucher, Bernhard Mayer

**Affiliations:** ^a^Water, Environment, Process Development and Analysis Division, Bureau de recherches géologiques et minières (BRGM), 45100 Orléans, France;; ^b^Rock-Water Interaction Group, Institute of Geological Sciences, University of Bern, CH-3012 Bern, Switzerland;; ^c^Department of Geoscience, University of Calgary, Calgary, AB, Canada T2N 1N4

**Keywords:** natural gas, radiolysis, hydrocarbons, carbon isotopes, shale

## Abstract

Natural gas is an important fossil energy source that has historically been produced from conventional hydrocarbon reservoirs. It has been interpreted to be of microbial, thermogenic, or, in specific contexts, abiotic origin. Since the beginning of the 21^st^ century, natural gas has been increasingly produced from unconventional hydrocarbon reservoirs including organic-rich shales. Here, we show, based on a careful interpretation of natural gas samples from numerous unconventional hydrocarbon reservoirs and results from recent irradiation experiments, that there is a previously overlooked source of natural gas that is generated by radiolysis of organic matter in shales. We demonstrate that radiolytic gas containing methane, ethane, and propane constitutes a significant end-member that can account for >25% of natural gas mixtures in major shale gas plays worldwide that have high organic matter and uranium contents. The consideration of radiolytic gas in natural gas mixtures provides alternative explanations for so-called carbon isotope reversals and suggests revised interpretations of some natural gas origins. We submit that considering natural gas of radiolytic origin as an additional component in uranium-bearing shale gas formations will lead to a more accurate determination of the origins of natural gas.

## Significance

Natural gas is a key fossil fuel as the world transitions away from coal toward less polluting energy sources in an attempt to minimize the impact of global climate change. Historically, the origin of natural gas produced from conventional reservoirs has been determined based on gas compositional data and stable isotope fingerprints of methane, ethane, and higher *n*-alkanes, revealing three dominant sources of natural gas: microbial, thermogenic, and abiotic. In our detailed synthesis of published natural gas data from a variety of unconventional hydrocarbon reservoirs worldwide, we demonstrate that there is a previously overlooked source of natural gas that is generated by radiolysis of organic matter in shales.

**Fig. 1.**
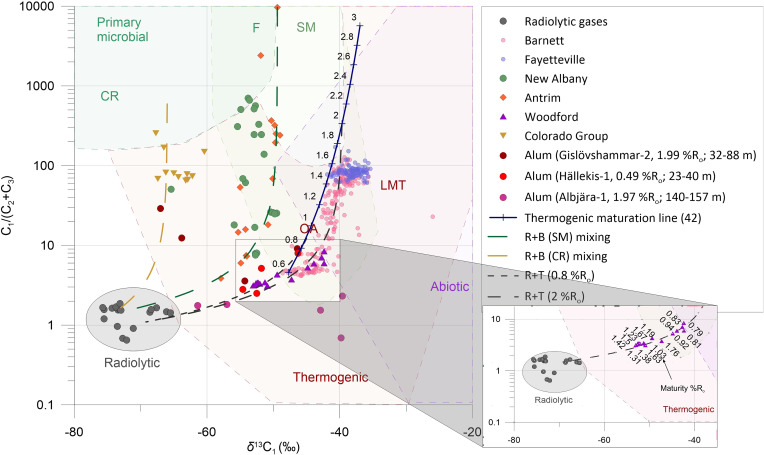
Revised Bernard plot after Milkov and Etiope (5). Dark blue line indicates a thermogenic maturation line according to ref. 6 with % R_o_ increasing from 0.6 to 3. Black dashed lines indicate mixing of radiolytic (R) and thermogenic (T) gas components; the green dashed line indicates the mixing of radiolytic gas with a mixture of primary biogenic gas (e.g., methyl type fermentation) and/or secondary microbial gas (both marked as B in the legend). The brown dashed line indicates mixing of radiogenic (R) and microbial gas derived by CO_2_ reduction. CR, CO_2_ reduction; F, methyl-type fermentation; SM, secondary microbial; OA, oil-associated (midmature) thermogenic gas; LMT, late mature thermogenic gas after Milkov et al. (1). (*Inset*) Data points from the Woodford Shale with maturities (7) increasing toward lower dryness values. Data are from radiolytic gases (8) and Barnett and Fayetteville (9), Antrim (10), New Albany (11), Woodford (7), Colorado Group (12), and Alum (13) shales.

Natural gas is extracted from conventional and unconventional hydrocarbon reservoirs to satisfy current energy demands. Three different origins of natural gas have been distinguished in previous literature including microbial, thermogenic, and abiotic (1). Some researchers also advocate for a low-temperature geocatalytic origin of some natural gases (ref. 2 and references therein). Microbial, thermogenic, and geocatalytic gases are derived from organic matter either by the action of microorganisms or due to elevated temperatures during burial of organic-rich sediments or through geocatalytic generation of nonmicrobial gases at low temperatures. Abiotic processes (3) do not involve organic matter but produce gases through gas–water–mineral interactions in the subsurface by reaction of native H_2_ with CO_2_ (4). The composition and isotopic signatures of natural gas components are frequently used to determine the origin and maturity of the natural gas (Fig. 1). Natural gases of microbial origin consist mostly of methane that is depleted in ^13^C (δ^13^C ranging between less than −90 and −50‰) (5). In contrast, thermogenic gases from shale gas reservoirs typically contain methane, ethane, propane, and higher *n*-alkanes with δ^13^C of methane varying between −75 and −20‰ (5) dependent on maturity. Geocatalytic gases mostly consist of methane with δ^13^C between −58 and −41‰ (14). Abiotic gases have a wide range of molecular compositions, and their methane is frequently enriched in ^13^C (δ^13^C ranging between −50 and +10‰) (5).

With the onset of the shale gas revolution early in the 21st century facilitated by horizontal drilling technologies combined with high-volume hydraulic fracturing, natural gas has been increasingly produced in recent years from unconventional hydrocarbon reservoirs such as shales with high organic matter content. Such shales are often associated with high contents of radioactive elements (15–17), and hence, the organic matter they contain is exposed to significant radiation doses over geologic time spans. Naturally occurring radioactive isotopes such as ^238^U, ^235^U, ^232^Th, ^230^Th, and ^40^K and their radioactive daughter products emit α- and β-particles and γ-rays that have penetration depths into the organic matter ranging from <100 µm for α-particles (18) and 1 to 5 mm for β-particles to >50 m for γ-rays (19). Potassium (K) and thorium (Th) are usually associated with detrital minerals. The concentration of radioactive ^40^K is too low in shales to produce significant irradiation of surrounding matter since ^40^K constitutes only 0.012% of all K isotopes (20), while concentrations of radioactive thorium can reach 20 ppm (e.g., refs. 21–23) and uranium (U) up to 2,600 ppm (24). Uranium is typically directly associated with organic matter in black shales (25–28), which will absorb most of the energy released during the U decay. For example, organic carbon in the Alum Shale in Europe with, on average, 100 ppm of U absorbed a 10^8^- to 10^9^-Gy radiation dose over the 500 Ma since its deposition (18, 29, 30).

High radiation doses can cause changes in the structure and properties of organic matter (31–35). For the fossil organic matter, kerogen, the most notable changes are an increase of aromaticity, of degree of condensation, and of vitrinite reflectance and a decrease in bitumen content (29, 36–42). Ionizing radiation causes polymerization, cross-linking, dealkylation, and aromatization of organic matter (29, 30, 43, 44) and has been shown to produce short-chain alkanes such as methane, ethane, and propane (8). Additionally, experimental irradiation of organic matter showed the importance of mineral surface area and a presence of clay minerals (44) in disintegration of organic matter and formation of radiolytic products including gases. Furthermore, during irradiation, ^−^H^·^ radicals form in large quantities (45), which might facilitate radiolytic formation of alkanes.

Laboratory-based irradiation experiments (8, 18) with organic matter and crude oils have revealed the formation of radiolytic gas that is mainly composed of H_2_ (56 to 96 vol. %), while around 2% of the newly formed gas is composed of methane, ethane, and propane with a linear positive relationship between the radiation dosage and the amount of radiolytic H_2_ and alkanes produced (19). These radiolytic hydrocarbons are derived from organic matter but neither through microbial nor through temperature-driven reactions, and they have been found to be depleted in ^13^C (δ^13^C of methane less than −65‰, δ^13^C of ethane less than −45‰, and δ^13^C of propane less than −37‰) (8).

We note that previous laboratory-based irradiation experiments using shales and fossil organic matter have not used α-particle irradiation, which mostly occurs in U-rich rocks. Thus, the findings and conclusions presented in this paper are based on the assumption that isotopic signatures of radiolytic gases produced during gamma-ray irradiation in laboratory experiments are equivalent to those resulting from alpha radiation in the geosphere. This is supported by similarities observed between irradiated organic matter in laboratory experiments and in nature. Experiments that used gamma rays from a ^60^Co source (18) demonstrated that irradiated organic matter in shales became slightly enriched in ^13^C requiring that the radiolytic gaseous products are depleted in ^13^C. The slight ^13^C enrichment of irradiated organic matter is also observed in natural U-rich rocks (37, 46–48), and thus, radiolytic hydrocarbons formed in such rocks are also expected to be depleted in ^13^C. This indirectly supports the notion that α-radiation in nature causes formation of ^13^C-depleted radiolytic gases in a very similar fashion to that of gamma radiation in laboratory experiments. However, laboratory data are currently scarce, and future experiments with α-particle irradiation of organic shales as well as controlled temperature parameters within the reaction chamber are needed.

This study investigates whether radiolytic methane, ethane, and propane (also referred to as “light alkanes” in the subsequent text) constitute a previously overlooked component of natural gas, especially in organic-rich shale gas plays. We demonstrate that light alkanes derived from the irradiation of kerogen and oil make a nonnegligible contribution to natural gas mixtures from unconventional hydrocarbon reservoirs. By using an isotopic maturation-mixing model on a large set of natural gas data, we quantify the effect of the admixture of light alkanes of radiolytic origin to gases of thermogenic and microbial origin. We also demonstrate that the resulting isotope signatures can lead to misinterpretation of gas origin and maturation levels, and we provide an alternative explanation of the so-called isotope reversals in natural gas from unconventional hydrocarbon reservoirs. We conclude that radiolytic gas derived from organic matter constitutes a previously not recognized type of natural gas that needs to be considered especially in organic-rich unconventional hydrocarbon reservoirs that frequently contain uranium (U) in substantial quantities (15, 16).

## Research Approach

A recent irradiation laboratory experiment conducted by Silva et al. (8) exposed crude oils from the Western Canadian Sedimentary Basin (Alberta heavy oil) and a Norwegian North Sea marine black oil (North Sea oil) initially containing no solution gas to gamma radiation doses (10^4^ to 10^6^ Gy at room temperature) resulting in the production of measurable quantities of radiolytic gases including methane, ethane, and propane with δ^13^C values of −71.7 ± 2.7‰, −49.1 ± 2.2‰, and −39.4 ± 1.9‰, respectively (8). We tested the hypothesis that radiolytic gas with such low δ^13^C values may constitute a significant end-member of natural gas mixtures from unconventional hydrocarbon reservoirs. To achieve this goal, we set up an isotopic mixing model that enabled us to quantify the isotope effects of admixture of variable amounts of radiolytic gases to thermogenic or microbial gases. The thermogenic gas component was calculated for various maturities with a model based on Faber et al. (6), as further explained in *Materials and Methods*. To compare our model calculations with data from natural gas samples, we used a large dataset of molecular and isotopic compositions of light hydrocarbons from selected organic-rich shales published by Sherwood et al. (49) supplemented by data from more recent publications (7, 13). The original samples and associated data were derived from Barnett and Fayetteville (9), Antrim (10), New Albany (11), Alum (13) (only sorbed gas data because free gas in the shallow parts of Alum Shale investigated in ref. 13 has microbial signatures), Colorado Group (12), and the Woodford shales (7). Most of the selected shale gas plays are characterized by low maturity and high radioactivity, with the Barnett and Fayetteville constituting examples of higher-maturity shales.

## Results and Discussion

### Identifying Radiolytic Gas Contributions in Natural Gas Sources.

Sources of natural gas have historically been identified by plots of carbon isotope ratios of methane versus gas dryness, which is the ratio of the concentrations of methane over the concentrations of the sum of higher *n*-alkanes such as ethane, propane, etc. [e.g., C_1_/(C_2_ + C_3_)] (50, 51). Fig. 1 shows such a plot according to refs. 1, 5 with the data for gas samples from all unconventional hydrocarbon reservoirs investigated in this study. In this diagram, microbial gas plots in the top left corner since δ^13^C values are typically low and negligible contents of ethane and propane result in a high gas dryness. In contrast, natural gases of thermogenic origin plot on the right side of the diagram along a trend of increasing maturity (see dark blue line in Fig. 1). Early mature thermogenic gases have δ^13^C values around −50‰ (5) and a low gas dryness, whereas an increase in the maturity of thermogenic gas reservoirs (as indicated by increasing vitrinite reflectance values R_o_) results in a trend of increasing δ^13^C values and elevated gas dryness values. In Fig. 1, we have also plotted radiolytic gases from Silva et al. (8) with low δ^13^C values of methane similar to those of microbial gas but a markedly lower gas dryness parameter around 1. Hence, these gases occupy a unique position in Fig. 1 that is distinct from the composition of other sources of natural gas in the geosphere (5).

**Fig. 2.**
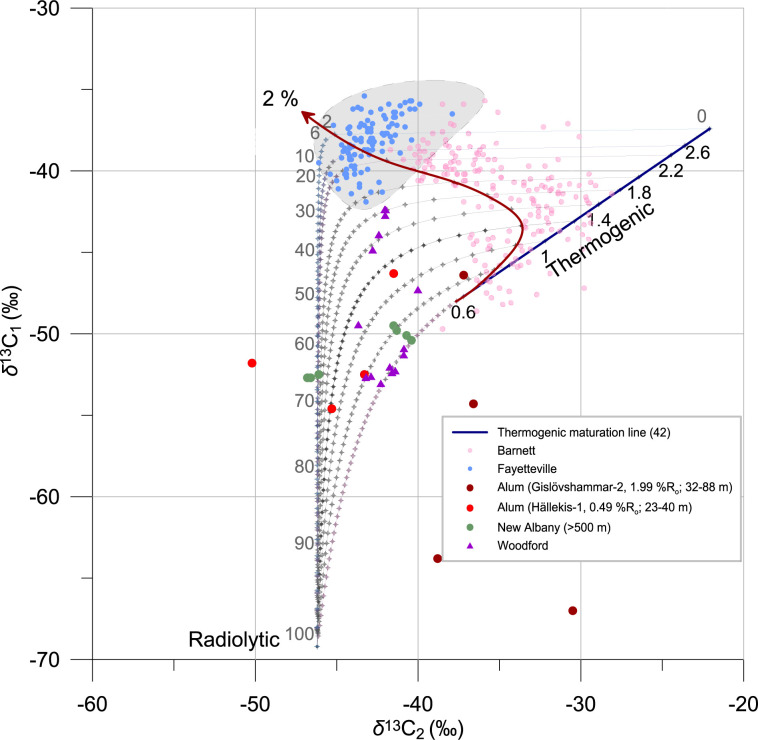
Two–end-member mixing model of radiolytic gas and thermogenic gases of different maturities. The dark blue line represents the maturation trend of kerogen II with numbers showing the maturation % R_o_ index after the model of Faber et al. (6). Black lines with dots are isotopic compositions of mixtures between radiolytic gas [produced from North Sea oil (8)] and thermogenic gases of different maturation stages. Numbers from 0 to 100 represent the fraction of radiolytic gas. The red arrow labeled 2% shows an admixture of a constant amount of radiolytic gas (arbitrary value of 2%) to thermogenic gas of different maturities. The gray region is the isotope reversal region (δ^13^C_1_ > δ^13^C_2_). Pink circles, Barnett Shale (9); blue circles, Fayetteville Shale (9); green circles, New Albany shale (11); violet triangles, Woodford Shale (7); red and dark red circles, Alum Shale (13).

Using our model, we have calculated two and three end-member mixing trend lines between the radiolytic gas end-member and 1) thermogenic gas (black dashed line in Fig. 1), 2) microbial gas derived by CO_2_ reduction (brown dashed line), and 3) a mixture of primary biogenic gas (e.g., methyl type fermentation) and/or secondary microbial gas (52) (with some addition of low-maturity thermogenic gas) (green dashed line). For clarity, green and brown mixing lines marked on Fig. 1 represent a mixture of radiolytic (R) and microbial (B) gases. However, for Antrim shales we tested three–end-member mixing of secondary microbial gas by adding small proportions (<10%) of low mature thermogenic gas (0.8% R_o_) and obtained mixing lines very close to the green two–end-member (SM-R) line on Fig. 1 (see also Dataset S1).

Inspection of Fig. 1 reveals that most of the plotted data points for natural gases from unconventional hydrocarbon reservoirs plot along these three mixing lines. This is an indication that radiolytic gas contributes to variable extents to the natural gas mixtures. For example, the Antrim Shale is a Devonian radioactive shale in North America, and the isotopic composition of its natural gas has been attributed to secondary microbial origin with some admixture of early mature thermogenic gases (11). The fact that the isotopic composition of natural gas samples plots near the mixing line between radiolytic and secondary microbial gases [with a small addition (<10%) of a thermogenic component] (green dashed line in Fig. 1) suggests that radiolytic gas is likely an additional third component of natural gas from the Antrim Shale. Also, gas samples from the low-maturity Colorado Group (R_o_ = 0.25%) (12) in AB, Canada, containing an abundance of radioactive elements (53) can be explained as a mixture of microbial gas derived from CO_2_ reduction and radiolytic gas that contains methane, ethane, and propane (brown dashed line in Fig. 1) rather than requiring a thermogenic gas component to explain the occurrence of ethane and propane (12).

We also found evidence of mixtures between radiolytic gas and low maturity thermogenic gas: samples from the low-maturity part of the Alum Shale (0.49% R_o_) (13) and the low-maturity to mid-maturity Woodford Shale (0.8 to 1.67% R_o_) (7, 54) are characterized by δ^13^C values of methane between −50 and −62‰ and gas dryness <5 (black dashed line in Fig. 1), which can only be explained by admixture of considerable proportions of radiolytic gas. Data for the Woodford Shale are of particular interest since they display a trend toward lower dryness with increasing maturity, while usually the dryness increases with increasing maturity (52, 55). Samples from this shale plot along a mixing line between radiolytic and thermogenic components, and more mature samples appear to be more affected by radiolytic gas components than less mature samples (Fig. 1, *Inset*). This is caused by the comparatively high abundances of ethane, propane, and butane in radiolytic gas, whereas in mature and highly mature thermogenic gases, methane is predominant. Hence, the admixture of even small amounts of radiolytic gases has a significant impact on the dryness of these gas mixtures and is readily detectable in Fig. 1.

Using Fig. 1 to identify radiolytic gas contributions to high-maturity thermogenic gases based on mixing calculations is challenging. Due to variability in natural gas maturity (solid dark blue line in Fig. 1), mixing of thermogenic gases of different maturities plots along a pattern that is rather similar to that of the mixing of thermogenic gases of higher maturities with radiolytic gases (black dashed line in Fig. 1 in OA area) as indicated for samples from the Barnett Shale. While this suggests that more refined approaches are required to quantify radiolytic contributions to natural gas mixtures with thermogenic components, the data shown in Fig. 1 strongly suggest the existence of previously overlooked natural gas contributions from radiolytic sources having a distinct chemical and isotopic composition in low-permeability hydrocarbon reservoirs.

### Quantifying Contributions of Radiolytic Gas Contributions.

To quantify contributions of radiolytic gas we used a two-source mixing model between radiolytic and thermogenic gas of different maturities based on δ^13^C values of methane and ethane shown in Fig. 2. The thick red arrow represents a hypothetical case where a conservative estimate of 2% radiolytic gas is mixed with thermogenic gases of different maturities. At the early stages of maturation, the admixture of a small amount of radiolytic gas to thermogenic gas of low maturity (<1% R_o_) will be almost indistinguishable from a normal maturation trend for thermogenic gas occurrences. For gases with maturity >1.8% R_o_, an admixture of small amounts of radiolytic gases results in marked shifts of the isotopic composition away from the maturation trend in the region of isotope reversal (gray area in Fig. 2). High-maturity thermogenic gases are increasingly dry (e.g., predominantly composed of methane), and hence, even small contributions of ^13^C-depleted ethane-rich radiolytic gas will decrease the δ^13^C of ethane in the gas mixture compared to the more ^13^C-enriched thermogenic ethane. In consequence, δ^13^C values of ethane (δ^13^C_2_) may be lower than those of methane (δ^13^C_1_), resulting in so-called “isotope reversals” (δ^13^C_1_ > δ^13^C_2_) frequently observed in high-maturity shale gas plays. Some high-maturity natural gas samples from Alum and Woodford shales plotting in the region of isotope reversal are likely explained by admixture of a radiolytic gas component. This reveals that isotope signatures of methane and especially ethane are highly sensitive to the admixture of small contributions of radiolytic gas to thermogenic natural gas occurrences, while providing a complementary explanation of so-called isotope reversals (δ^13^C_1_ > δ^13^C_2_) in high-maturity unconventional hydrocarbon reservoirs.

Similarly, samples from the Woodford Shale and from the deep sections (>500 m) of the New Albany Shale can be interpreted as a mixture of radiolytic and thermogenic gases. Natural gas samples from the low- to medium-maturity Woodford Shale (0.6 to 1.8% R_o_) (7, 56) plot on two mixing lines: early thermogenic gas mixed with 20 to 30% of radiolytic gas and thermogenic gas of medium maturity mixed with ∼10% of radiolytic gas (Fig. 2). The occurrence of a considerable contribution of radiolytic gas in samples from the Woodford Shale explains previous contradictory observations of gas compositions consistent with low-maturity shales coupled with higher *n*-alkanes (e.g., pentane and hexane) and biomarkers suggesting a thermogenic gas end-member with medium to high maturity [see Abrams and Thomas (56) and references therein]. The New Albany Shale has low to medium maturity (0.5 to 1.5% R_o_), and its U concentration associated with amorphous organic matter is ca. 550 ppm (27). Natural gas produced from the New Albany Shale plots on the mixing line between thermogenic and radiolytic gas in Fig. 2, indicating a considerable (>25%) contribution of radiolytic gases.

It is important to note that admixture of radiolytic gases containing methane, ethane, and propane with low δ^13^C values will decrease the δ^13^C values of light alkanes in gas mixtures between radiolytic and thermogenic gas. Not considering this radiolytic gas contribution can result in misinterpretations of the gas mixture as thermogenic gas of lower maturity or even as microbial gas. Furthermore, ^13^C-depleted radiolytic ethane constitutes a previously not considered explanation of the comparatively high abundance of ethane in some biogenic gases and in shallow aquifers overlying thick sedimentary shale units (57).

So far, only one experiment (18) measured the amount of radiolytic gases produced during irradiation of shale and reported production of up to 4 µmol of methane per gram of organic carbon in shales at an irradiation dose of 8.85 × 10^6^ Gy. The experimental dose was three orders of magnitude lower than estimates for the Cambrian (>500 Ma) Alum Shales throughout the Phanerozoic. Four µmol/g of methane is only three times lower than the measured production of thermogenic hydrocarbons at a maturation of <1% R_o_ (58). Therefore, it is plausible that at least 25% of methane in geologically old early mature shales is of radiolytic origin. Judging from the presented mixing model and available data for natural gas samples from the Antrim, New Albany, Alum, and Woodford shales, radiolytic hydrocarbons appear to constitute a significant portion (>20%) of gas mixtures, consistent with the above-mentioned experimental data (18, 58). For mature shales, the admixture of radiolytic methane is, however, comparatively small compared to the predominantly thermogenic gas contribution. For gas samples from highly mature reservoir rocks, detection of small radiolytic gas contributions may be possible using ethane and propane concentrations as demonstrated by samples from the Woodford Shale. Data shown in Fig. 2 indicate that radiolytic gas has negligible contributions (<2%) in samples from the Barnett and Fayetteville shale gas reservoirs, presumably due to their comparatively low U contents.

## Conclusions

Uranium-rich and organic-rich shales provide all the ingredients for the generation of radiolytic gases composed of H_2_, CO_2_, and hydrocarbon gases due to their radioactivity (7) and the presence of clay minerals (9). Our investigation of compositional and isotopic data for natural gas samples from a wide variety of unconventional hydrocarbon reservoirs strongly suggests that radiolytic gases containing methane, ethane, and propane with comparatively low δ^13^C values constitute a previously overlooked component of many natural gas mixtures. In early mature shales, the contribution of radiolytic gases can exceed 25%, and ignoring its presence can result in misinterpretations of the gas origin, including erroneous claims of microbial gas occurrences or identification of thermogenic gases of too low maturity. In mature shales the admixture of a small amount of radiolytic component to high-maturity thermogenic natural gas represents an alternative mechanism that can explain carbon isotope reversals. We recommend a reassessment of previous gas source attributions in cases where radiolytic gas contributions may be significant as a result of elevated radioactivity associated with organic matter in shales to enable a more accurate assessment of the origin of natural gas mixtures required to sustain future energy demands from fossil fuels.

So far, only gas compositions and δ^13^C values of light radiolytic hydrocarbons have been published for laboratory experiments. However, CO_2_ and H_2_ are produced in much larger quantities (up to 96% of all radiolytic gases) than hydrocarbons. Future work should also determine the isotopic signatures of these gases including δ^13^C_CO2_, δD_H2_, and δD_CH4_, which could provide additional parameters for understanding the genesis of hydrocarbon gases (3). Clumped isotopes (Δ18 values of methane, Δ47 of CO_2_, and ΔDD of H_2_) and position-specific isotope analyses may be used to calculate formation temperatures (59–62) and might help to elucidate gas formation mechanisms in future laboratory experiments on radiolytic hydrocarbons.

## Materials and Methods

This study is based on previously published data of molecular and isotopic compositions of light hydrocarbons from selected unconventional hydrocarbon plays derived from the database compiled by Sherwood et al. (49) complemented by data from more recent publications (7, 13). The original samples were derived from Barnett and Fayetteville shales (9), Antrim Shale (10), New Albany Shale (11), Alum Shale (13), Colorado Group Shale (12), and the Woodford Shale (7). Most of the selected shale gas plays are characterized by low maturity and high radioactivity, conditions favorable for the generation of radiolytic *n*-alkanes. Data for sorbed gas not displaying microbial signatures from the Alum Shale are included due to high radioactivity of this shale. The well-studied Barnett and Fayetteville shales are included as representatives of high-maturity hydrocarbon plays, although the radioactivity of these shales is only moderate.

The composition and carbon isotope ratios of *n*-alkanes such as methane, ethane, propane, etc., of gas samples were used in our model that combines a carbon isotope maturity model (6) and an isotopic mixing model (63). For the mixing model, we used several gas end-members: radiolytic, thermogenic of different maturity, microbial gas derived by CO_2_ reduction, and a mixture of primary biogenic gas (e.g., methyl type fermentation) and/or secondary microbial gas.

The molecular and isotopic compositions of the radiolytic gases are from Silva et al. (8). The authors exposed dead crude oils from the Western Canadian Sedimentary Basin (Alberta heavy oil) and a Norwegian North Sea marine black oil (North Sea oil), which initially contained no solution gas, to gamma radiation doses from 0.05 to 10 MGy at a dose rate of 13.05 kGy/h at room temperature. The experiment resulted in the production of measurable quantities of radiolytic gases including methane, ethane, propane, and *n*-butane. The average molecular compositions and δ^13^C ranges are as follows:

• Alberta heavy oil:
  o Methane (C_1_) 56.5%, from −75.7 to −67.6‰;
  o Ethane (C_2_) 23.5%, from −51.9 to −50.2‰;
  o Propane (C_3_) 10.7%, from −41.9 to −40.2‰;
• North Sea oil:
  o Methane (C_1_) 46.6%, from −72.7 to −66.1‰;
  o Ethane (C_2_) 19.9%, from −47.2 to −45.4‰;
  o Propane (C_3_) 11.6%, from −38.6 to −33.3‰.

For our mixing model calculations we assumed that radiolytic gases produced from kerogen have similarly low δ^13^C values to the radiolytic gases described by Silva et al. (8), which is consistent with earlier studies reporting that irradiated kerogen became slightly enriched in ^13^C with increasing radiation doses (36, 43, 46, 47). The molecular and isotopic compositions of thermogenic gas components were calculated with a model based on Faber et al. (6), equations 1–6, and isotopic mixing was calculated following equation **7** of Berner and Faber (63). Maturation of thermogenic gas was modeled for a marine kerogen II precursor with initial δ^13^C_1_ = −33‰, δ^13^C_2_ = −30‰, and δ^13^C_3_ = −26‰. δ^13^C values were calculated following equations 1–6 in ref. 6 assuming maturation with R_o_ (vitrinite reflectance) values between 0.6 and 3.0%. For example, for low maturity of 0.6% R_o_, δ^13^C values for methane, ethane, and propane were −47.7, −37.2, and −30.3‰ with molecular compositions of 79.64, 12.35, and 5.00%, respectively. For a high maturity of 3.0% R_o_, δ^13^C values for methane, ethane, and propane were −37.0, −21.4, and −15.7‰ with concentrations of 99.98, 0.016, and 0.001% (Dataset S1). The molecular and isotopic compositions of thermogenic gases derived from the model of Faber et al. (6) have exponential relationships (thick blue line in Fig. 1 with numbers from 0.6 to 3 referring to % R_o_). Isotopic compositions of thermogenic gases depend on multiple factors, including the initial C isotope ratio and compositional variations of kerogen from shale to shale. Thus, we use this model to show a general maturation trend and consider maturity estimations derived from Faber et al. (6) qualitatively but not quantitatively. We consider gases from reservoir rocks with maturity <0.8% R_o_ as early mature, those from reservoir rocks ranging from 0.8 to 1.5% R_o_ likely derived from oil-associated sources, and dry gases derived from reservoir rocks with >2% R_o_ as mature thermogenic gases (64).

A sample from the Antrim Shale (10) with the highest C_1_/(C_2_ + C_3_) representing a mixture of primary microbial gas (e.g., methyl type fermentation) and secondary microbial gas is used as a third end-member with the following composition and C isotope ratios: δ^13^C_1_ = −49.4‰, C_1_ = 96.56 vol. %, δ^13^C_2_ = −34.3‰, C_2_ = 0.01 vol. %, and C_3_ absent.

## Supplementary Material

Supplementary File

## Data Availability

All data used for this work are from previously published papers (5, 7–13, 49).

## References

[1] A. V. Milkov, M. Faiz, G. Etiope, Geochemistry of shale gases from around the world: Composition, origins, isotope reversals and rollovers, and implications for the exploration of shale plays. Org. Geochem. 143, 103997 (2020).

[2] X. Ma , Methane generation from low-maturity coals and shale source rocks at low temperatures (80–120°C) over 14–38 months. Org. Geochem. 155, 104224 (2021).

[3] G. Etiope, B. Sherwood Lollar, Abiotic methane on Earth. Rev. Geophys. 51, 276–299 (2013).

[4] G. Etiope, M. Schoell, Abiotic gas: Atypical, but not rare. Elements 10, 291–296 (2014).

[5] A. V. Milkov, G. Etiope, Revised genetic diagrams for natural gases based on a global dataset of >20,000 samples. Org. Geochem. 125, 109–120 (2018).

[6] E. Faber, M. Schmidt, A. Feyzullayev, Geochemical hydrocarbon exploration—Insights from stable isotope models. Oil Gas Eur. Mag. 41, 93–98 (2015).

[7] C. Liu, P. Liu, G. P. McGovern, J. Horita, Molecular and intramolecular isotope geochemistry of natural gases from the Woodford Shale, Arkoma Basin, Oklahoma. Geochim. Cosmochim. Acta 255, 188–204 (2019).

[8] R. C. Silva , Radiolysis as a source of ^13^C depleted natural gases in the geosphere. Org. Geochem. 138, 103911 (2019).

[9] J. Zumberge, K. Ferworn, S. Brown, Isotopic reversal (‘rollover’) in shale gases produced from the Mississippian Barnett and Fayetteville formations. Mar. Pet. Geol. 31, 43–52 (2012).

[10] A. M. Martini , Microbial production and modification of gases in sedimentary basins: A geochemical case study from a Devonian shale gas play, Michigan basin. Am. Assoc. Pet. Geol. Bull. 87, 1355–1375 (2003).

[11] J. C. McIntosh, L. M. Walter, A. M. Martini, Pleistocene recharge to midcontinent basins: Effects on salinity structure and microbial gas generation. Geochim. Cosmochim. Acta 66, 1681–1700 (2002).

[12] D. Rowe, K. Muehlenbachs, Isotopic fingerprints of shallow gases in the Western Canadian sedimentary basin: Tools for remediation of leaking heavy oil wells. Org. Geochem. 30, 861–871 (1999).

[13] N. H. Schovsbo, A. T. Nielsen, Generation and origin of natural gas in Lower Palaeozoic shales from southern Sweden. Geol. Surv. Denmark Greenl. Bull. 38, 37–40 (2017).

[14] L. Wei , Catalytic generation of methane at 60–100 °C and 0.1–300 MPa from source rocks containing kerogen Types I, II, and III. Geochim. Cosmochim. Acta 231, 88–116 (2018).

[15] V. E. Swanson, Geology and Geochemistry of Uranium in Marine Black Shales. A Review. (Geological Survey, US Government Printing Office, Washington, DC, 1961), pp. 1–110.

[16] M. P. Ketris, Y. E. Yudovich, Estimations of Clarkes for carbonaceous biolithes: World averages for trace element contents in black shales and coals. Int. J. Coal Geol. 78, 135–148 (2009).

[17] H.-M. Schulz , The Furongian to Lower Ordovician Alum Shale Formation in conventional and unconventional petroleum systems in the Baltic Basin—A review. Earth Sci. Rev. 218, 103674 (2021).

[18] M. D. Lewan, G. F. Ulmishek, W. Harrison, F. Schreiner, Gamma ^60^Co-irradiation of organic matter in the phosphoria retort shale. Geochim. Cosmochim. Acta 55, 1051–1063 (1991).

[19] H. Wang , Oil generation from the immature organic matter after artificial neutron irradiation. Energy Fuels 34, 1276–1287 (2020).

[20] M. O. Naumenko, K. Mezger, T. F. Nägler, I. M. Villa, High precision determination of the terrestrial ^40^K abundance. Geochim. Cosmochim. Acta 122, 353–362 (2013).

[21] A. P. Ruotsala, Mineralogy of Antrim Shale, Michigan (Dow Chemical Co., Midland, MI, 1980).

[22] X. Qiu , Major, trace and platinum-group element geochemistry of the Upper Triassic nonmarine hot shales in the Ordos basin, Central China. Appl. Geochem. 53, 42–52 (2015).

[23] R. Karma, G. Parslow, Sedimentology and geochemistry of the Bakken Formation (Devonian-Mississippian) in southern Saskatchewan. Summary Invest. 89, 141–147 (1989).

[24] A. Lecomte, M. Cathelineau, R. Michels, C. Peiffert, M. Brouand, Uranium mineralization in the Alum Shale Formation (Sweden): Evolution of a U-rich marine black shale from sedimentation to metamorphism. Ore Geol. Rev. 88, 71–98 (2017).

[25] R. Finch, T. Murakami, “Systematics and paragenesis of uranium minerals” in Uranium, P. C. Burns, R. J. Finch, Eds. (*Reviews in Mineralogy*, De Gruyter, 1999), vol. 38, pp. 91–180.

[26] S. Lüning, S. Kolonic, Uranium spectral gamma‐ray response as a proxy for organic richness in black shales: Applicability and limitations. J. Pet. Geol. 26, 153–174 (2003).

[27] B. Liu, M. Mastalerz, J. Schieber, J. Teng, Association of uranium with macerals in marine black shales: Insights from the Upper Devonian New Albany Shale, Illinois Basin. Int. J. Coal Geol. 217, 103351 (2020).

[28] J. G. Lerat , Metals and radionuclides (MaR) in the Alum Shale of Denmark: Identification of MaR-bearing phases for the better management of hydraulic fracturing waters. J. Nat. Gas Sci. Eng. 53, 139–152 (2018).

[29] M. D. Lewan, B. Buchardt, Irradiation of organic matter by uranium decay in the Alum Shale, Sweden. Geochim. Cosmochim. Acta 53, 1307–1322 (1989).

[30] S. Yang , Geological alteration of organic macromolecules by irradiation: Implication for organic matter occurrence on Mars. Geology 48, 713–717 (2020).

[31] C. S. Spirakis, The roles of organic matter in the formation of uranium deposits in sedimentary rocks. Ore Geol. Rev. 11, 53–69 (1996).

[32] S. Ortaboy, G. Atun, Kinetics and equilibrium modeling of uranium (VI) sorption by bituminous shale from aqueous solution. Ann. Nucl. Energy 73, 345–354 (2014).

[33] H. Sanei, H. I. Petersen, N. H. Schovsbo, C. Jiang, M. E. Goodsite, Petrographic and geochemical composition of kerogen in the Furongian (U. Cambrian) Alum Shale, central Sweden: Reflections on the petroleum generation potential. Int. J. Coal Geol. 132, 158–169 (2014).

[34] P. Landais, Organic geochemistry of sedimentary uranium ore deposits. Ore Geol. Rev. 11, 33–51 (1996).

[35] V. S. Ivanov, Radiation Chemistry of Polymers (VSP, Utrecht, 1992).

[36] J. Dahl, R. Hallberg, I. Kaplan, Effects of irradiation from uranium decay on extractable organic matter in the Alum Shales of Sweden. Org. Geochem. 12, 559–571 (1988).

[37] J. Dahl, R. Hallberg, I. Kaplan, The effects of radioactive decay of uranium on elemental and isotopic ratios of Alum Shale kerogen. Appl. Geochem. 3, 583–589 (1988).

[38] F. Gauthier-Lafaye, F. Weber, “Uranium-hydrocarbon association in Francevillian uranium ore deposits, Lower Proterozoic of Gabon” in Bitumens in Ore Deposits, J. Parnell, H. Kucha, P. Landais, Eds. (Springer, 1993), pp. 276–286.

[39] B. Nagy , Role of organic matter in the Proterozoic Oklo natural fission reactors, Gabon, Africa. Geology 21, 655–658 (1993).

[40] H.-M. Schulz, S. Yang, E. Panova, A. Bechtel, The role of Pleistocene meltwater-controlled uranium leaching in assessing irradiation-induced alteration of organic matter and petroleum potential in the Tremadocian Koporie Formation (Western Russia). Geochim. Cosmochim. Acta 245, 133–153 (2019).

[41] S. Y. Yang , On the changing petroleum generation properties of Alum Shale over geological time caused by uranium irradiation. Geochim. Cosmochim. Acta 229, 20–35 (2018).

[42] C. M. B. Jaraula , Radiolytic alteration of biopolymers in the Mulga Rock (Australia) uranium deposit. Appl. Geochem. 52, 97–108 (2015).

[43] S. Y. Yang, H. M. Schulz, N. Schovsbo, S. Mayanna, The organic geochemistry of “Kolm”, a unique analogue for the understanding of molecular changes after significant uranium irradiation. Int. J. Coal Geol. 209, 89–93 (2019).

[44] C. J. Boreham, J. B. Davies, Carbon and hydrogen isotopes of the wet gases produced by gamma-ray-induced polymerisation of methane: Insights into radiogenic mechanism and natural gas formation. Radiat. Phys. Chem. 168, 108546 (2020).

[45] A. C. Fox, J. L. Eigenbrode, K. H. Freeman, Radiolysis of macromolecular organic material in Mars‐relevant mineral matrices. J. Geophys. Res. 124, 3257–3266 (2019).

[46] F. Zhang, Y. Jiao, L. Wu, H. Rong, B. Zang, Changes in physicochemical properties of organic matter by uranium irradiation: A case study from the Ordos Basin in China. J. Environ. Radioact. 211, 106105 (2020). 31739226 10.1016/j.jenvrad.2019.106105

[47] J. S. Leventhal, C. N. Threlkeld, Carbon-13/carbon-12 isotope fractionation of organic matter associated with uranium ores induced by alpha irradiation. Science 202, 430–432 (1978). 17836756 10.1126/science.202.4366.430

[48] R. W. Court, M. A. Sephton, J. Parnell, I. Gilmour, The alteration of organic matter in response to ionising irradiation: Chemical trends and implications for extraterrestrial sample analysis. Geochim. Cosmochim. Acta 70, 1020–1039 (2006).

[49] O. A. Sherwood, S. Schwietzke, V. A. Arling, G. Etiope, Global inventory of gas geochemistry data from fossil fuel, microbial and burning sources, version 2017. Earth Syst. Sci. Data **9**, 639–656 (2017).

[50] B. B. Bernard, J. M. Brooks, W. M. Sackett, Natural gas seepage in the Gulf of Mexico. Earth and Planetary Science Letters 31, 48–54 (1976).

[51] A. V. Milkov, S. Schwietzke, G. Allen, O. A. Sherwood, G. Etiope, Using global isotopic data to constrain the role of shale gas production in recent increases in atmospheric methane. Sci. Rep. 10, 4199 (2020). 32144290 10.1038/s41598-020-61035-w PMC7060170

[52] M. J. Whiticar, Carbon and hydrogen isotope systematics of bacterial formation and oxidation of methane. Chem. Geol. 161, 291–314 (1999).

[53] K. S. Nielsen, C. J. Schröder-Adams, D. A. Leckie, A new stratigraphic framework for the Upper Colorado Group (Cretaceous) in southern Alberta and southwestern Saskatchewan, Canada. Bull. Can. Pet. Geol. 51, 304–346 (2003).

[54] T. Wang, “An organic geochemical study of Woodford Shale and Woodford-Mississippian tight oil from Central Oklahoma,” PhD dissertation, University of Oklahoma, Norman, OK (2016), p. 299.

[55] C. R. Evans, M. A. Rogers, N. J. L. Bailey, Evolution and alteration of petroleum in western Canada. Chem. Geol. 8, 147–170 (1971).

[56] M. A. Abrams, D. Thomas, Geochemical evaluation of oil and gas samples from the Upper Devonian and Mississippian reservoirs Southern Anadarko Basin Oklahoma and its implication for the Woodford Shale unconventional play. Mar. Pet. Geol. 112, 104043 (2020).

[57] P. Humez , Redox controls on methane formation, migration and fate in shallow aquifers. Hydrol. Earth Syst. Sci. 20, 2759–2777 (2016).

[58] R. J. Hill, Y. Tang, I. R. Kaplan, Insights into oil cracking based on laboratory experiments. Org. Geochem. 34, 1651–1672 (2003).

[59] D. A. Stolper , The utility of methane clumped isotopes to constrain the origins of methane in natural gas accumulations. Geol. Soc. Spec. Publ. 468, 23–52 (2018).

[60] I. Prokhorov, T. Kluge, C. Janssen, Optical clumped isotope thermometry of carbon dioxide. Sci. Rep. 9, 4765 (2019). 30886173 10.1038/s41598-019-40750-z PMC6423234

[61] M. E. Popa, D. Paul, C. Janssen, T. Röckmann, H_2_ clumped isotope measurements at natural isotopic abundances. Rapid Commun. Mass Spectrom. 33, 239–251 (2019). 30378194 10.1002/rcm.8323 PMC6590658

[62] A. Gilbert, K. Yamada, K. Suda, Y. Ueno, N. Yoshida, Measurement of position-specific ^13^C isotopic composition of propane at the nanomole level. Geochim. Cosmochim. Acta 177, 205–216 (2016).

[63] U. Berner, E. Faber, Maturity related mixing model for methane, ethane and propane, based on carbon isotopes, Proceedings of the 13^th^ International Meeting on Organic Geochemistry. Org. Geochem. 13, 67–72 (1988).

[64] J. Cesar, M. Nightingale, V. Becker, B. Mayer, Stable carbon isotope systematics of methane, ethane and propane from low-permeability hydrocarbon reservoirs. Chem. Geol. 558, 119907 (2020).

